# Corrigendum: Targeting Bcl-2 Proteins in Acute Myeloid Leukemia

**DOI:** 10.3389/fonc.2020.632775

**Published:** 2020-12-17

**Authors:** Yunxiong Wei, Yaqing Cao, Rui Sun, Lin Cheng, Xia Xiong, Xin Jin, Xiaoyuan He, Wenyi Lu, Mingfeng Zhao

**Affiliations:** ^1^The First Central Clinical College of Tianjin Medical University, Tianjin, China; ^2^Nankai University School of Medicine, Tianjin, China; ^3^Department of Hematology, Tianjin First Central Hospital, Tianjin, China

**Keywords:** B cell lymphoma 2, AML—acute myeloid leukemia, venetoclax (ABT-199), intrinsic apoptosis, Bcl-2 protein

In the published article, there was an error regarding the affiliation(s) for Mingfeng Zhao. Instead of “Mingfeng Zhao ^1,3^”, it should be “Mingfeng Zhao ^3^”.

The authors apologize for this error and state that this does not change the scientific conclusions of the article in any way. The original article has been updated.

